# Supplementary figures and images for: Study on Potato Bud Cultivation Techniques in a Greenhouse in Spring
Source: Plants (Basel). 2023 Oct 12;12(20):3545. doi: 10.3390/plants12203545 (PMC10610138; doi:10.3390/plants12203545)

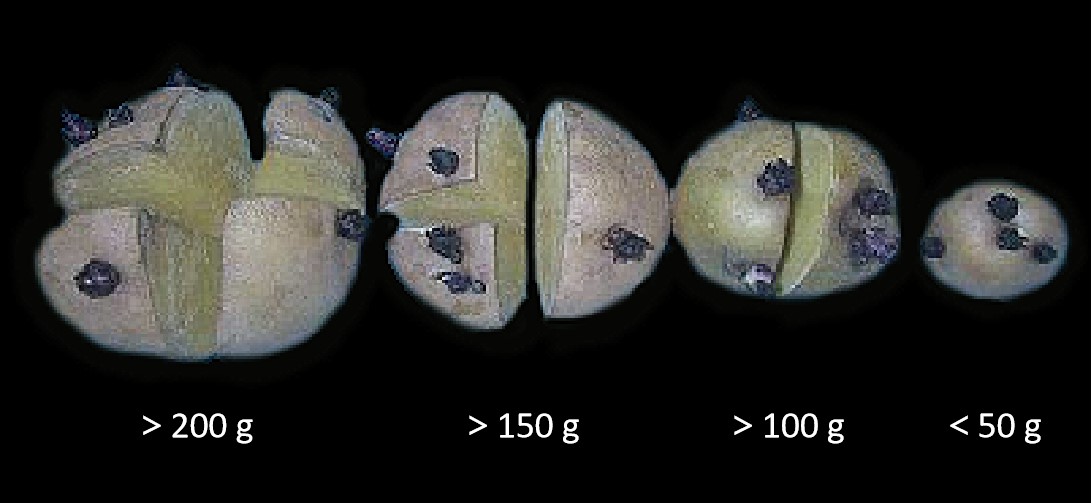

Supplement: Supplementary file 1 [file plants-12-03545-s001.zip › figure s1.jpg]

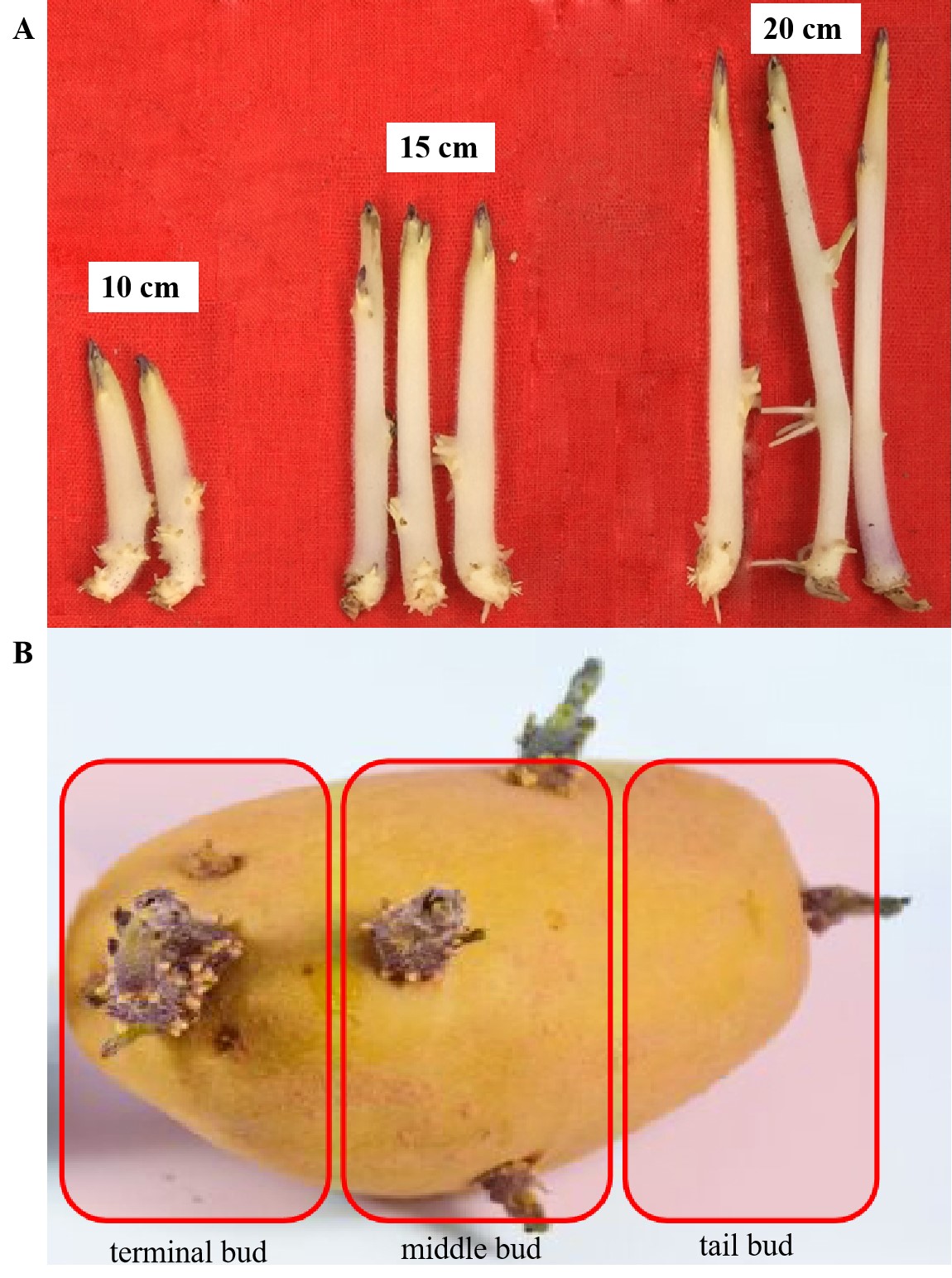

Supplement: Supplementary file 1 [file plants-12-03545-s001.zip › figure S2.jpg]
